# Comparing segmentations by applying randomization techniques

**DOI:** 10.1186/1471-2105-8-171

**Published:** 2007-05-23

**Authors:** Niina Haiminen, Heikki Mannila, Evimaria Terzi

**Affiliations:** 1 HIIT Basic Research Unit, Department of Computer Science, P.O.Box 68, FI-00014 University of Helsinki, Finland; 2 Laboratory of Computer and Information Science, Helsinki University of Technology, FI-02015 TKK, Finland

## Abstract

**Background:**

There exist many segmentation techniques for genomic sequences, and the segmentations can also be based on many different biological features. We show how to evaluate and compare the quality of segmentations obtained by different techniques and alternative biological features.

**Results:**

We apply randomization techniques for evaluating the quality of a given segmentation. Our example applications include isochore detection and the discovery of coding-noncoding structure. We obtain segmentations of relevant sequences by applying different techniques, and use alternative features to segment on. We show that some of the obtained segmentations are very similar to the underlying true segmentations, and this similarity is statistically significant. For some other segmentations, we show that equally good results are likely to appear by chance.

**Conclusion:**

We introduce a framework for evaluating segmentation quality, and demonstrate its use on two examples of segmental genomic structures. We transform the process of quality evaluation from simply viewing the segmentations, to obtaining *p*-values denoting significance of segmentation similarity.

## Background

Segmental structure of various scales exists in genomic sequences. Many evolutionary and genetic mechanisms leading to variation in DNA operate on segments of the genome (duplications and inversions of segments, recombinations). Furthermore, eukaryotic chromosomes consist of alternating regions of gene-rich and gene-poor regions. A gene-rich region can be further decomposed into non-coding segments, segments that contain regulatory information, and genes, which in turn consist of introns and exons. Also, remnants of viral or microbial inserts in a genome form a type of segmental structure.

There are many types of features with which one can segment the sequences. For any given technique, there may exist alternative biological features to segment on. For example, if the goal is to identify coding and noncoding segments in a sequence, one may study the distribution of three-letter words (codons) along the sequence to determine where to set the segment boundaries. An alternative would be, for example, to segment on the frequency of nucleotides in each third position, as done in [1].

There are also many possible segmentation techniques for discovering the segmental structure in DNA sequences. The techniques include recursive segmentation methods [1-7], Bayesian methods [8,9], hidden Markov models [10-13], and wavelet analysis [14], among others. For a review of various approaches, see [15].

If a reliable annotation of the underlying segmental structure exists, it is of interest to find out which feature set and segmentation method give a result closest to the true segmental structure. This can give insight into the biological process that is responsible for creating and maintaining the segmental structure. Furthermore, the segmentation technique and feature set that is the best on an annotated sequence may also yield results close to true segmental structure on unannotated data.

In order to find differences between segmentations one has to define a notion of similarity or distance between them. In this paper we describe the distance between two segmentations *P *and *Q *by two numbers – conditional entropy of *P *given *Q*, and vice versa. For a   similar approach to comparing clusterings see Meilă [26]. Conditional   entropy is an information theoretic measure that quantifies the amount   of information that one segmentation gives about the other. The sum of   these two conditional entropies also defines the entropy distance   between the two segmentations. Ideally, we want both terms in the sum   to be small, instead of only requiring their sum to be small.  We give   an example of how this makes a difference when comparing segmentations.   By using this measure we can easily rank a set of segmentations with   respect to their distance from the underlying true segmentation, if one   is known.

The sum of these two conditional entropies also defines the entropy distance between the two segmentations. Ideally, we want both terms in the sum to be small, instead of only requiring their sum to be small. We give an example of how this makes a difference when comparing segmentations. By using this measure we can easily rank a set of segmentations with respect to their distance from the underlying true segmentation, if one is known.

Knowing the best segmentation technique and feature set for a given sequence is still not enough. Namely, one candidate segmentation could be better than the other candidates, but all could still be quite far from the true one. That is, we want to find out if the best result is in some sense significant. The problem of deciding segmentation significance has been addressed before in the case where the sequence data itself is known [9]. Our approach does not rely on the sequence data, since it takes as input only the set of segmentations we want to evaluate. Our technique is therefore more general, and also applies to cases where the segmentations are obtained by using alternative biological features. To our knowledge, the issue of significance of segmentation similarity has not been considered before.

We test the significance of segmentation similarity by generating random segmentations and computing the distances between the underlying true segmentation and the randomized segmentations. If a random segmentation is about as close to the true one as our candidate segmentation is, then the agreement between the true segmentation and our candidate one is not very interesting. Note that in our randomization approach we do not make any assumptions regarding the properties of a good segmentation, but only consider the values of conditional entropies summarizing the similarity between two segmentations. To define a randomization procedure, we have to specify the class of segmentations from which we sample random elements. In this paper we use two classes of segmentation: (a) segmentations that have a given *k *number of segments, and (b) segmentations that have the same number of segments and the same segment-length distribution as the true segmentation.

We apply this randomization technique to examples on coding-noncoding structure and to isochore detection. The results show that the small distances obtained by some segmentation techniques and biological features are indeed significant, while for others, the obtained segmentations are only as similar to the ground truth as a majority of the randomly generated segmentations.

## Results and discussion

In this section we show the results of our randomization techniques for evaluating the discovered segmentations in two examples of genomic sequences for which a segmental structure is already known.

### Example 1: Coding-noncoding structure

Discovering the locations of genes in a DNA sequence is an important task to which computational methods give different predictions. In this example, we evaluate the closeness of different segmentation results to known gene boundaries.

We used a dataset consisting of a 25 kb region of bacterium *Rickettsia prowazekii *[16] (positions 535 – 560 kb), containing 13 coding segments. The correct number of segments in the data is 25 (13 genes separated by 12 noncoding segments [GenBank:AJ235269]). We denote the correct underlying segmentation into coding and noncoding regions by *T*.

We applied the encoding scheme described in [1] to transform the DNA sequence into a 12-dimensional signal. This encoding of the sequence captures the codon usage, see [1] and references therein. The segmentation techniques we applied are the entropic segmentation (*E*) by Bernaola-Galván et al. [1] and the least squares segmentation method (*L*). In the entropic segmentation the input is split recursively until no more significant splits can be found. The optimal least squares segmentation with *k *segments is computed with a dynamic-programming algorithm [17]. Note that the entropic segmentation decides on the number of segments, while the least-squares method takes the number of segments as input. The entropic segmentation method found 19 segments (at 99% significance level) for this dataset, outputting segmentation *E*_*cd*_. The least squares method for *k *= 25 output segmentation *L*_*cd*_.

We additionally applied the least squares method on six other features: frequencies of 2-letter words (AA,AC,...,TT), 1-letter words (A,C,G,T), and each nucleotide separately (A;C;G;T). We denote these features by {2,1, *A*, *C*, *G*, *T*} respectively. The sequences we constructed in such a way correspond to a 16-dimensional, a 4-dimensional and four 1-dimensional signals. Each point in these sequences corresponds to the frequency of a feature in an 10 bp window. We divided the sequence into 2500 non-overlapping windows.

**Figure 1:**
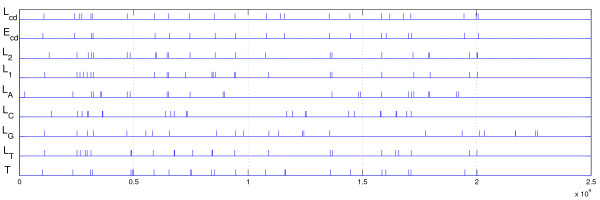
**Rickettsia prowazekii segmentations**. *Rickettsia prowazekii *segmentations and true segmentation *T*. *E*_*cd*_: entropic segmentation method with codon frequencies [1]. *L*_*f*_: least-squares segmentation with features *f*; *f *∈ {*cd*, 2, 1, *A*, *C*, *G*, *T*} indicate the codon feature, frequencies of 2-letter words, 1-letter words, and frequency of A, C, G, or T, respectively.

The obtained segmentations are shown in Figure 1, along with the known boundaries *T*. We use *E*_*f *_and *L*_*f *_to denote the output of the entropic and the dynamic-programming algorithm on a sequence obtained using feature *f*. From the figure its hard to conclude which one of the output segmentations is the closest to *T *and whether the same measure of agreement could arise by chance.

**Figure 2:**
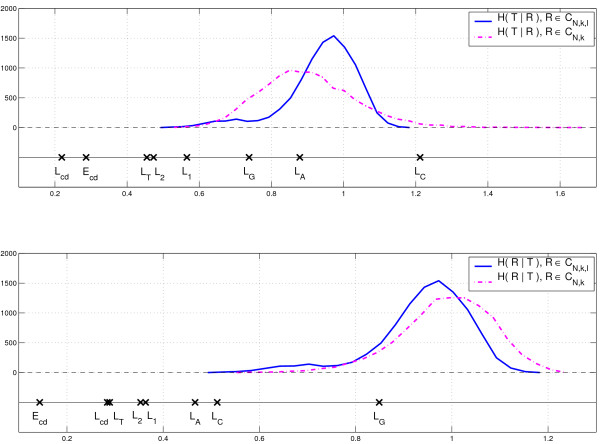
**Randomization of Rickettsia prowazekii segmentations**. Randomization of *Rickettsia prowazekii *segmentations: conditional entropies. *E*_*cd*_: entropic segmentation method with codon frequencies. *L*_*f*_: least-squares segmentation with features *f*; *f *∈ {*cd*, 2, 1, *A*, *C*, *G*, *T*} indicate the codon feature, frequencies of 2-letter words, 1-letter words, and frequency of A, C, G, or T, respectively.

To evaluate the significance of our findings we applied the randomization tests described in the Methods section. We generated random segmentations from classes CN,k
 MathType@MTEF@5@5@+=feaafiart1ev1aaatCvAUfKttLearuWrP9MDH5MBPbIqV92AaeXatLxBI9gBaebbnrfifHhDYfgasaacH8akY=wiFfYdH8Gipec8Eeeu0xXdbba9frFj0=OqFfea0dXdd9vqai=hGuQ8kuc9pgc9s8qqaq=dirpe0xb9q8qiLsFr0=vr0=vr0dc8meaabaqaciaacaGaaeqabaqabeGadaaakeaaruWrPXgBtfMBZbaceaGae83qam0aaSbaaSqaaGqaciab+5eaoHqaaiab9XcaSiab+TgaRbqabaaaaa@348B@ and CN,k,ℓ
 MathType@MTEF@5@5@+=feaafiart1ev1aaatCvAUfKttLearuWrP9MDH5MBPbIqV92AaeXatLxBI9gBaebbnrfifHhDYfgasaacH8akY=wiFfYdH8Gipec8Eeeu0xXdbba9frFj0=OqFfea0dXdd9vqai=hGuQ8kuc9pgc9s8qqaq=dirpe0xb9q8qiLsFr0=vr0=vr0dc8meaabaqaciaacaGaaeqabaqabeGadaaakeaaruWrPXgBtfMBZbaceaGae83qam0aaSbaaSqaaGqaciab+5eaoHqaaiab9XcaSiab+TgaRjab+XcaSiabloriSbqabaaaaa@3698@. The distributions of conditional entropies *H *(*R*|*T*) and *H *(*T*|*R*) for *R *∈ CN,k
 MathType@MTEF@5@5@+=feaafiart1ev1aaatCvAUfKttLearuWrP9MDH5MBPbIqV92AaeXatLxBI9gBaebbnrfifHhDYfgasaacH8akY=wiFfYdH8Gipec8Eeeu0xXdbba9frFj0=OqFfea0dXdd9vqai=hGuQ8kuc9pgc9s8qqaq=dirpe0xb9q8qiLsFr0=vr0=vr0dc8meaabaqaciaacaGaaeqabaqabeGadaaakeaaruWrPXgBtfMBZbaceaGae83qam0aaSbaaSqaaGqaciab+5eaoHqaaiab9XcaSiab+TgaRbqabaaaaa@348B@ for and *R *∈ CN,k,ℓ
 MathType@MTEF@5@5@+=feaafiart1ev1aaatCvAUfKttLearuWrP9MDH5MBPbIqV92AaeXatLxBI9gBaebbnrfifHhDYfgasaacH8akY=wiFfYdH8Gipec8Eeeu0xXdbba9frFj0=OqFfea0dXdd9vqai=hGuQ8kuc9pgc9s8qqaq=dirpe0xb9q8qiLsFr0=vr0=vr0dc8meaabaqaciaacaGaaeqabaqabeGadaaakeaaruWrPXgBtfMBZbaceaGae83qam0aaSbaaSqaaGqaciab+5eaoHqaaiab9XcaSiab+TgaRjab+XcaSiabloriSbqabaaaaa@3698@ in 10,000 randomizations are shown in Figure 2. The conditional entropies and the empirical *p*-values are given in Table 1. The values *p*_*ℓ *_and *p*_*k *_correspond to the *p*-values obtained by *ℓ *and *k*-randomizations respectively. From the figure and the table we see that segmentations *L*_*cd*_, *E*_*cd*_, *L*_2 _and *L*_1 _and *L*_*T *_are all closer to the ground truth segmentation than would be expected by chance. For any of the above segmentations, the values of the two conditional entropies (see Table 1) are much lower than those appearing in the two randomization tests. On the other hand, segmentations *L*_*A *_and *L*_*C *_should not be considered similar to *T*. Although *H *(*L*_*A*_|*T*) and *H *(*L*_*C*_|*T*) are small, the values of *H *(*T*|*L*_*A*_) and *H *(*T*|*L*_*C*_) do occur often in the randomization. Thus segmentations *L*_*A *_and *L*_*C  *_fail one of the two randomization tests and are not considered to be close to the ground truth. In this example, the choice between CN,k
 MathType@MTEF@5@5@+=feaafiart1ev1aaatCvAUfKttLearuWrP9MDH5MBPbIqV92AaeXatLxBI9gBaebbnrfifHhDYfgasaacH8akY=wiFfYdH8Gipec8Eeeu0xXdbba9frFj0=OqFfea0dXdd9vqai=hGuQ8kuc9pgc9s8qqaq=dirpe0xb9q8qiLsFr0=vr0=vr0dc8meaabaqaciaacaGaaeqabaqabeGadaaakeaaruWrPXgBtfMBZbaceaGae83qam0aaSbaaSqaaGqaciab+5eaoHqaaiab9XcaSiab+TgaRbqabaaaaa@348B@ and CN,k,ℓ
 MathType@MTEF@5@5@+=feaafiart1ev1aaatCvAUfKttLearuWrP9MDH5MBPbIqV92AaeXatLxBI9gBaebbnrfifHhDYfgasaacH8akY=wiFfYdH8Gipec8Eeeu0xXdbba9frFj0=OqFfea0dXdd9vqai=hGuQ8kuc9pgc9s8qqaq=dirpe0xb9q8qiLsFr0=vr0=vr0dc8meaabaqaciaacaGaaeqabaqabeGadaaakeaaruWrPXgBtfMBZbaceaGae83qam0aaSbaaSqaaGqaciab+5eaoHqaaiab9XcaSiab+TgaRjab+XcaSiabloriSbqabaaaaa@3698@ does not make a difference in deciding the significance.

**Table 1:** Conditional entropies and their significances for segmentations on coding-noncoding data

*P*	*H *(*T*|*P*)	*p*_*ℓ*_	*p*_*k*_	*H *(*P*|*T*)	*p*_*ℓ*_	*p*_*k*_
*L*_*cd*_	0.219	0	0	0.284	0	0
*E*_*cd*_	0.286	0	0	0.143	0	0
*L*_2_	0.473	0	0	0.353	0	0
*L*_1_	0.566	0.0018	0.0005	0.364	0	0
*L*_*A*_	0.879	0.1642	0.4219	0.466	0	0
*L*_*C*_	1.212	1	0.9770	0.512	0	0.0001
*L*_*G*_	0.738	0.0522	0.0732	0.849	0.1195	0.0661
*L*_*T*_	0.455	0	0	0.289	0	0

Conditional entropies and their significances for segmentations on coding-noncoding data. *E*_*cd*_: entropic segmentation with codon features. *L*_*f*_: least-squares segmentation with features *f*; *f *∈ {*cd*, 2, 1, *A*, *C*, *G*, *T*} indicate the codon feature, frequencies of 2-letter words, 1-letter words, and frequency of A, C, G, or T, respectively, *p*_*ℓ*_: the fraction of segmentations from CN,k,ℓ
 MathType@MTEF@5@5@+=feaafiart1ev1aaatCvAUfKttLearuWrP9MDH5MBPbIqV92AaeXatLxBI9gBaebbnrfifHhDYfgasaacH8akY=wiFfYdH8Gipec8Eeeu0xXdbba9frFj0=OqFfea0dXdd9vqai=hGuQ8kuc9pgc9s8qqaq=dirpe0xb9q8qiLsFr0=vr0=vr0dc8meaabaqaciaacaGaaeqabaqabeGadaaakeaaruWrPXgBtfMBZbaceaGae83qam0aaSbaaSqaaGqaciab+5eaoHqaaiab9XcaSiab+TgaRjab+XcaSiabloriSbqabaaaaa@3698@ with a smaller value of the conditional entropy in 10,000 randomizations; *p*_*k*_: the fraction of segmentations from CN,k
 MathType@MTEF@5@5@+=feaafiart1ev1aaatCvAUfKttLearuWrP9MDH5MBPbIqV92AaeXatLxBI9gBaebbnrfifHhDYfgasaacH8akY=wiFfYdH8Gipec8Eeeu0xXdbba9frFj0=OqFfea0dXdd9vqai=hGuQ8kuc9pgc9s8qqaq=dirpe0xb9q8qiLsFr0=vr0=vr0dc8meaabaqaciaacaGaaeqabaqabeGadaaakeaaruWrPXgBtfMBZbaceaGae83qam0aaSbaaSqaaGqaciab+5eaoHqaaiab9XcaSiab+TgaRbqabaaaaa@348B@ with a smaller value of the conditional entropy in 10,000 randomizations.

### Example 2: Isochore structure

Isochores are large (>> 300 kb) DNA segments fairly homogeneous in their guanine and cytosine (G+C) content. Exactly defining isochore borders in the human genome remains an open problem, but different computational approaches exist. Isochores are discussed in biological literature already since [18]. In our experiments we used a dataset consisting of the 100 Mb short arm telomeric region of human chromosome 1 ([19]). Unlike in the previous example, there is no well defined biological annotation of the segmental (isochore) structure of this genomic region. We consider as the ground truth segmentation *T *results by Costantini et al. [20] and alternatively results by IsoFinder [7]. These results differ considerably in their segment number (*k *= 114 vs *k *= 1305), reflecting genomic structures at different granularities. We also study segmentations on the major histocompatibility (MHC) region in chromosome 6, for which some biologically validated isochore boundaries are known. To generate candidate segmentations, we use the least squares dynamic programming algorithm (*L*) described in the previous example, with the same number of segments as the in respective *T*.

**Figure 3:**
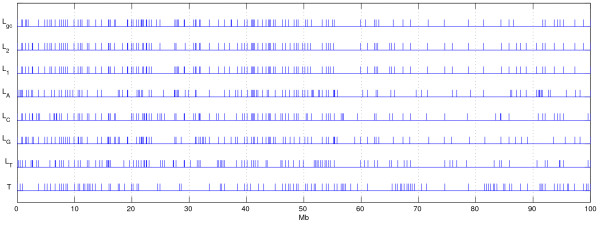
**Isochore segmentations**. Isochore segmentations of Chromosome 1 100 Mb region with *k *= 114 and reference segmentation *T *from [20]. *L*_*f*_: least-squares segmentation with features *f*; *f *∈ {*gc*, 2, 1, *A*, *C*, *G*, *T*} indicate frequencies of G+C, 2-letter words, 1-letter words, and frequency of A, C, G, T, or G+C, respectively.

**Figure 4:**
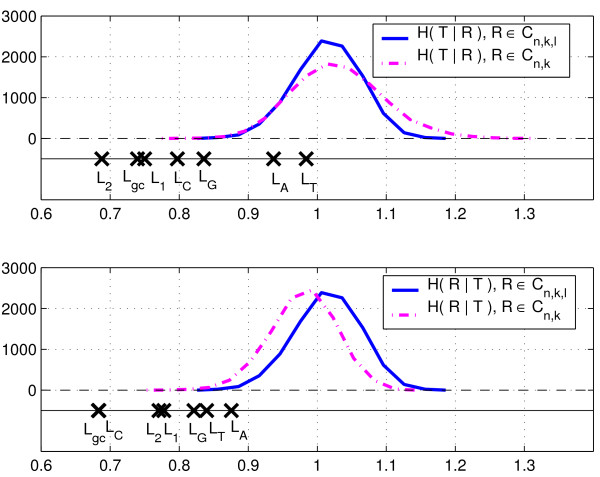
**Randomization of isochore segmentations**. Randomization of Chromosome 1 isochore segmentations with *k *= 114: conditional entropies. *L*_*f*_: least-squares segmentation with features *f*; *f *∈ {*gc*, 2, 1, *A*, *C*, *G*, *T*} indicate frequencies of G+C, 2-letter words, 1-letter words, and frequency of A, C, G, or T respectively.

First, we consider the segmentation results by Costantini et al. [20] as the ground truth segmentation *T*, in which there are 114 segments. For generating candidate segmentations, we aggregated the data into 100 kb windows (obtaining 1000 data points). We use *L*_*gc *_to denote the output segmentation of the least squares method on the signal summarizing the G+C content of the sequence. Besides the G+C content we also applied the least-squares method on the six features we considered in the previous section. Therefore, we again constructed the sequences on features {2, 1, *A*, *C*, *G*, *T*}, that correspond to the frequencies of 2-letter words (AA,AC,...,TT), 1-letter words (A,C,G,T), and each nucleotide separately (A;C;G;T). The segmentations are shown in Figure 3, along with the chosen reference segmentation *T*. Again, it is not immediately clear which one of the segmentations is closest to *T*. The conditional entropies and the corresponding *p*-values for *ℓ *and *k*-randomizations are shown in Table 2. Figure 4 shows the distributions of conditional entropies *H *(*R*|*T*) and *H *(*T*|*R*) for *R *∈ CN,k
 MathType@MTEF@5@5@+=feaafiart1ev1aaatCvAUfKttLearuWrP9MDH5MBPbIqV92AaeXatLxBI9gBaebbnrfifHhDYfgasaacH8akY=wiFfYdH8Gipec8Eeeu0xXdbba9frFj0=OqFfea0dXdd9vqai=hGuQ8kuc9pgc9s8qqaq=dirpe0xb9q8qiLsFr0=vr0=vr0dc8meaabaqaciaacaGaaeqabaqabeGadaaakeaaruWrPXgBtfMBZbaceaGae83qam0aaSbaaSqaaGqaciab+5eaoHqaaiab9XcaSiab+TgaRbqabaaaaa@348B@ for and *R *∈ CN,k,ℓ
 MathType@MTEF@5@5@+=feaafiart1ev1aaatCvAUfKttLearuWrP9MDH5MBPbIqV92AaeXatLxBI9gBaebbnrfifHhDYfgasaacH8akY=wiFfYdH8Gipec8Eeeu0xXdbba9frFj0=OqFfea0dXdd9vqai=hGuQ8kuc9pgc9s8qqaq=dirpe0xb9q8qiLsFr0=vr0=vr0dc8meaabaqaciaacaGaaeqabaqabeGadaaakeaaruWrPXgBtfMBZbaceaGae83qam0aaSbaaSqaaGqaciab+5eaoHqaaiab9XcaSiab+TgaRjab+XcaSiabloriSbqabaaaaa@3698@ in 10,000 randomizations. Segmentations *L*_*gc*_, *L*_2_, *L*_1_, *L*_*C *_and *L*_*G *_are significantly close to *T*. This is somehow expected given the importance of *G *and *C *concentration in the isochore structure. Note that segmentation *IF*_*gc*_, as a high entropy segmentation, has a significantly small value of *H *(*T*|*IF*_*gc*_), but the value of *H *(*IF*_*gc*_|*T*) is by far larger that the entropy of *H *(*R*|*T*) for any random segmentation *R*.

**Table 2:** Conditional entropies and their significances for isochore segmentations with 114 segments

*P*	*H *(*T*|*P*)	*p*_*ℓ*_	*p*_*k*_	*H *(*P*|*T*)	*p*_*ℓ*_	*p*_*k*_
*L*_*gc*_	0.740	0	0	0.683	0	0
*L*_2_	0.688	0	0	0.771	0	0
*L*_1_	0.750	0	0	0.778	0	0
*L*_*A*_	0.937	0.0352	0.0389	0.876	0.0010	0.0090
*L*_*C*_	0.797	0	0	0.684	0	0
*L*_*G*_	0.836	0.0002	0.0004	0.821	0.0001	0.0005
*L*_*T*_	0.984	0.1758	0.1748	0.840	0.0002	0.0010

Conditional entropies and entropy distances for segmentations on isochore structure, for the ground truth segmentation from [20] with *k *= 114. *L*_*f*_: least-squares segmentation with features *f*; *f *∈ {*gc*, 2, 1, *A*, *C*, *G*, *T*} indicate frequencies of G+C, 2-letter words, 1-letter words, and frequency of A, C, G, or T, respectively, *p*_*ℓ*_: the fraction of segmentations from CN,k,ℓ
 MathType@MTEF@5@5@+=feaafiart1ev1aaatCvAUfKttLearuWrP9MDH5MBPbIqV92AaeXatLxBI9gBaebbnrfifHhDYfgasaacH8akY=wiFfYdH8Gipec8Eeeu0xXdbba9frFj0=OqFfea0dXdd9vqai=hGuQ8kuc9pgc9s8qqaq=dirpe0xb9q8qiLsFr0=vr0=vr0dc8meaabaqaciaacaGaaeqabaqabeGadaaakeaaruWrPXgBtfMBZbaceaGae83qam0aaSbaaSqaaGqaciab+5eaoHqaaiab9XcaSiab+TgaRjab+XcaSiabloriSbqabaaaaa@3698@ with a smaller value of the conditional entropy in 10,000 randomizations; *p*_*k*_: the fraction of segmentations from CN,k
 MathType@MTEF@5@5@+=feaafiart1ev1aaatCvAUfKttLearuWrP9MDH5MBPbIqV92AaeXatLxBI9gBaebbnrfifHhDYfgasaacH8akY=wiFfYdH8Gipec8Eeeu0xXdbba9frFj0=OqFfea0dXdd9vqai=hGuQ8kuc9pgc9s8qqaq=dirpe0xb9q8qiLsFr0=vr0=vr0dc8meaabaqaciaacaGaaeqabaqabeGadaaakeaaruWrPXgBtfMBZbaceaGae83qam0aaSbaaSqaaGqaciab+5eaoHqaaiab9XcaSiab+TgaRbqabaaaaa@348B@ with a smaller value of the conditional entropy in 10,000 randomizations.

Next we consider IsoFinder [7] results as the ground truth segmentation *T*, with *k *= 1305. We generated least squares candidate segmentations *L*_*f *_with this *k *and the same features *f *as in the previous case, the only difference being that here the sequence was aggregated into 10 kb windows (obtaining 10,000 data points). The segmentations are shown in Additional File 1, along with the chosen reference segmentation *T*. It is not clear from the figure which of the segmentations, if any, are close to *T*. The distributions of conditional entropies *H *(*R*|*T*) and *H *(*T*|*R*) for *R *∈ CN,k
 MathType@MTEF@5@5@+=feaafiart1ev1aaatCvAUfKttLearuWrP9MDH5MBPbIqV92AaeXatLxBI9gBaebbnrfifHhDYfgasaacH8akY=wiFfYdH8Gipec8Eeeu0xXdbba9frFj0=OqFfea0dXdd9vqai=hGuQ8kuc9pgc9s8qqaq=dirpe0xb9q8qiLsFr0=vr0=vr0dc8meaabaqaciaacaGaaeqabaqabeGadaaakeaaruWrPXgBtfMBZbaceaGae83qam0aaSbaaSqaaGqaciab+5eaoHqaaiab9XcaSiab+TgaRbqabaaaaa@348B@ for and *R *∈ CN,k,ℓ
 MathType@MTEF@5@5@+=feaafiart1ev1aaatCvAUfKttLearuWrP9MDH5MBPbIqV92AaeXatLxBI9gBaebbnrfifHhDYfgasaacH8akY=wiFfYdH8Gipec8Eeeu0xXdbba9frFj0=OqFfea0dXdd9vqai=hGuQ8kuc9pgc9s8qqaq=dirpe0xb9q8qiLsFr0=vr0=vr0dc8meaabaqaciaacaGaaeqabaqabeGadaaakeaaruWrPXgBtfMBZbaceaGae83qam0aaSbaaSqaaGqaciab+5eaoHqaaiab9XcaSiab+TgaRjab+XcaSiabloriSbqabaaaaa@3698@ in 10,000 randomizations are also shown in the Additional File 1. In this case, the significance results depend on the choice of *k*-versus *ℓ*-randomization: if the former is chosen, all the candidate segmentations are significantly close to *T*. This means that the least squares segmentations are indeed significantly closer to *T *than segmentations with randomly assigned *k *boundaries. In the case of *ℓ*-randomization, we find that *H *(*R*|*T*) <* H *(*L*_*f*_|*T*) for all our candidate segmentations *L*_*f*_. Thus all segmentations *L*_*f *_are far from *T*. The reason is that the segment length distribution of *T *is far from the distribution for any *L*_*f*_, and this prevents the candidate segmentations from being significantly close to *T*.

We also study the major histocompatibility (MHC) region in chromosome 6 ([21]). The 3.67 Mb region contains two experimentally validated isochore structures [22]. The known MHC isochore boundaries are around 1.8 Mb, 2.5 Mb and 3.4 Mb. We studied predictions from wavelet multiresolution analysis by Wen and Zhang [14] (*k *= 7), results by Costantini et al. [20] (*k *= 6), and predictions by the tool IsoFinder [23] (*k *= 8), choosing in turn each one of these as the ground truth and thus performing three randomization experiments. We used least squares segmentations (with *k *= 7) as alternative candidate segmentations (as discussed earlier, with window size 100 kb). The segmentations and results from the randomization tests are shown in Additional File 2. The randomization tests show that all segmentations, except in some cases those for features *A *and *T*, are significantly close to the chosen ground truth segmentation. In particular, all the ground truth segmentations are found to be significantly close to each other.

## Conclusion

In biological sequence analysis, there exist situations where many alternative segmentations for the underlying biological structure are proposed. We give a framework for evaluating the quality of results produced by different segmentation methods. Our approach also applies to cases where the segmentations are obtained by using alternative biological features, as we base our analysis only on the segment boundaries. Applicable segmentation distance measures and randomization tests are discussed, and results are shown for two applications of segmenting genomic data.

## Methods

### Comparing segmentations

We first give some intuition for our segmentation-comparison method and provide some basic definitions. Consider a sequence *S *with length |*S*| = *N *and a segmentation *P *of *S*. The segmentation partitions the sequence into *k *non-overlapping and contiguous intervals that span the whole sequence and they are called *segments*. A segmentation *P *with *k *segments can be fully defined using (*k *+ 1) segment boundaries *p*_0_,...,*p*_*k*_, where *p*_*i *_∈ *S*, *p*_*i *_<*p*_*i*+1 _for every *i*, and *p*_0 _= 0 and *p*_*k *_= *N*. The *i*-th segment of *P*, denoted by p¯i
 MathType@MTEF@5@5@+=feaafiart1ev1aaatCvAUfKttLearuWrP9MDH5MBPbIqV92AaeXatLxBI9gBaebbnrfifHhDYfgasaacH8akY=wiFfYdH8Gipec8Eeeu0xXdbba9frFj0=OqFfea0dXdd9vqai=hGuQ8kuc9pgc9s8qqaq=dirpe0xb9q8qiLsFr0=vr0=vr0dc8meaabaqaciaacaGaaeqabaqabeGadaaakeaacuWGWbaCgaqeamaaBaaaleaacqWGPbqAaeqaaaaa@2FB4@, is defined to be the interval p¯i
 MathType@MTEF@5@5@+=feaafiart1ev1aaatCvAUfKttLearuWrP9MDH5MBPbIqV92AaeXatLxBI9gBaebbnrfifHhDYfgasaacH8akY=wiFfYdH8Gipec8Eeeu0xXdbba9frFj0=OqFfea0dXdd9vqai=hGuQ8kuc9pgc9s8qqaq=dirpe0xb9q8qiLsFr0=vr0=vr0dc8meaabaqaciaacaGaaeqabaqabeGadaaakeaacuWGWbaCgaqeamaaBaaaleaacqWGPbqAaeqaaaaa@2FB4@ = (*p*_*i*-1_, *p*_*i*_]. Each segment p¯i
 MathType@MTEF@5@5@+=feaafiart1ev1aaatCvAUfKttLearuWrP9MDH5MBPbIqV92AaeXatLxBI9gBaebbnrfifHhDYfgasaacH8akY=wiFfYdH8Gipec8Eeeu0xXdbba9frFj0=OqFfea0dXdd9vqai=hGuQ8kuc9pgc9s8qqaq=dirpe0xb9q8qiLsFr0=vr0=vr0dc8meaabaqaciaacaGaaeqabaqabeGadaaakeaacuWGWbaCgaqeamaaBaaaleaacqWGPbqAaeqaaaaa@2FB4@ consists of |p¯i
 MathType@MTEF@5@5@+=feaafiart1ev1aaatCvAUfKttLearuWrP9MDH5MBPbIqV92AaeXatLxBI9gBaebbnrfifHhDYfgasaacH8akY=wiFfYdH8Gipec8Eeeu0xXdbba9frFj0=OqFfea0dXdd9vqai=hGuQ8kuc9pgc9s8qqaq=dirpe0xb9q8qiLsFr0=vr0=vr0dc8meaabaqaciaacaGaaeqabaqabeGadaaakeaacuWGWbaCgaqeamaaBaaaleaacqWGPbqAaeqaaaaa@2FB4@| points that correspond to the length of the segment.

Consider now segmentation *P *of consisting of *k *segments P={p¯1,...,p¯k}
 MathType@MTEF@5@5@+=feaafiart1ev1aaatCvAUfKttLearuWrP9MDH5MBPbIqV92AaeXatLxBI9gBaebbnrfifHhDYfgasaacH8akY=wiFfYdH8Gipec8Eeeu0xXdbba9frFj0=OqFfea0dXdd9vqai=hGuQ8kuc9pgc9s8qqaq=dirpe0xb9q8qiLsFr0=vr0=vr0dc8meaabaqaciaacaGaaeqabaqabeGadaaakeaacqWGqbaucqGH9aqpcqGG7bWEcuWGWbaCgaqeamaaBaaaleaacqaIXaqmaeqaaOGaeiilaWIaeiOla4IaeiOla4IaeiOla4IaeiilaWIafmiCaaNbaebadaWgaaWcbaGaem4AaSgabeaakiabc2ha9baa@3C04@. If we randomly pick a point *x *on the sequence, then the probability that *x *∈ p¯i
 MathType@MTEF@5@5@+=feaafiart1ev1aaatCvAUfKttLearuWrP9MDH5MBPbIqV92AaeXatLxBI9gBaebbnrfifHhDYfgasaacH8akY=wiFfYdH8Gipec8Eeeu0xXdbba9frFj0=OqFfea0dXdd9vqai=hGuQ8kuc9pgc9s8qqaq=dirpe0xb9q8qiLsFr0=vr0=vr0dc8meaabaqaciaacaGaaeqabaqabeGadaaakeaacuWGWbaCgaqeamaaBaaaleaacqWGPbqAaeqaaaaa@2FB4@ is Pr⁡(p¯i)=|p¯i|N
 MathType@MTEF@5@5@+=feaafiart1ev1aaatCvAUfKttLearuWrP9MDH5MBPbIqV92AaeXatLxBI9gBaebbnrfifHhDYfgasaacH8akY=wiFfYdH8Gipec8Eeeu0xXdbba9frFj0=OqFfea0dXdd9vqai=hGuQ8kuc9pgc9s8qqaq=dirpe0xb9q8qiLsFr0=vr0=vr0dc8meaabaqaciaacaGaaeqabaqabeGadaaakeaacyGGqbaucqGGYbGCcqGGOaakcuWGWbaCgaqeamaaBaaaleaacqWGPbqAaeqaaOGaeiykaKIaeyypa0ZaaSaaaeaacqGG8baFcuWGWbaCgaqeamaaBaaaleaacqWGPbqAaeqaaOGaeiiFaWhabaGaemOta4eaaaaa@3C53@. Since the segments cover the whole sequence we have ∑p¯i∈PPr⁡(p¯i)=1
 MathType@MTEF@5@5@+=feaafiart1ev1aaatCvAUfKttLearuWrP9MDH5MBPbIqV92AaeXatLxBI9gBaebbnrfifHhDYfgasaacH8akY=wiFfYdH8Gipec8Eeeu0xXdbba9frFj0=OqFfea0dXdd9vqai=hGuQ8kuc9pgc9s8qqaq=dirpe0xb9q8qiLsFr0=vr0=vr0dc8meaabaqaciaacaGaaeqabaqabeGadaaakeaadaaeqaqaaiGbccfaqjabckhaYjabcIcaOaWcbaGafmiCaaNbaebadaWgaaadbaGaemyAaKgabeaaliabgIGiolabdcfaqbqab0GaeyyeIuoakiqbdchaWzaaraWaaSbaaSqaaiabdMgaPbqabaGccqGGPaqkcqGH9aqpcqaIXaqmaaa@3DAA@. Therefore, we can define the *entropy *of a segmentation *P *to be

H(P)=−∑i=1kPr⁡(p¯i)log⁡Pr⁡(p¯i).
 MathType@MTEF@5@5@+=feaafiart1ev1aaatCvAUfKttLearuWrP9MDH5MBPbIqV92AaeXatLxBI9gBaebbnrfifHhDYfgasaacH8akY=wiFfYdH8Gipec8Eeeu0xXdbba9frFj0=OqFfea0dXdd9vqai=hGuQ8kuc9pgc9s8qqaq=dirpe0xb9q8qiLsFr0=vr0=vr0dc8meaabaqaciaacaGaaeqabaqabeGadaaakeaacqWGibascqGGOaakcqWGqbaucqGGPaqkcqGH9aqpcqGHsisldaaeWbqaaiGbccfaqjabckhaYjabcIcaOiqbdchaWzaaraWaaSbaaSqaaiabdMgaPbqabaGccqGGPaqkaSqaaiabdMgaPjabg2da9iabigdaXaqaaiabdUgaRbqdcqGHris5aOGagiiBaWMaei4Ba8Maei4zaCMagiiuaaLaeiOCaiNaeiikaGIafmiCaaNbaebadaWgaaWcbaGaemyAaKgabeaakiabcMcaPiabc6caUaaa@4D45@

The maximum value that the entropy of a segmentation can have is log *N*.

Consider now a pair of segmentations *P *and *Q *of sequence *S*. Assume that *P *and *Q *have *k*_*p *_and *k*_*q *_segments, respectively, such that *P *= {p¯1,...,p¯kp}
 MathType@MTEF@5@5@+=feaafiart1ev1aaatCvAUfKttLearuWrP9MDH5MBPbIqV92AaeXatLxBI9gBaebbnrfifHhDYfgasaacH8akY=wiFfYdH8Gipec8Eeeu0xXdbba9frFj0=OqFfea0dXdd9vqai=hGuQ8kuc9pgc9s8qqaq=dirpe0xb9q8qiLsFr0=vr0=vr0dc8meaabaqaciaacaGaaeqabaqabeGadaaakeaacqGG7bWEcuWGWbaCgaqeamaaBaaaleaacqaIXaqmaeqaaOGaeiilaWIaeiOla4IaeiOla4IaeiOla4IaeiilaWIafmiCaaNbaebadaWgaaWcbaGaem4AaS2aaSbaaWqaaiabdchaWbqabaaaleqaaOGaeiyFa0haaa@3B76@ and *Q *= {q¯1,...,q¯kq}
 MathType@MTEF@5@5@+=feaafiart1ev1aaatCvAUfKttLearuWrP9MDH5MBPbIqV92AaeXatLxBI9gBaebbnrfifHhDYfgasaacH8akY=wiFfYdH8Gipec8Eeeu0xXdbba9frFj0=OqFfea0dXdd9vqai=hGuQ8kuc9pgc9s8qqaq=dirpe0xb9q8qiLsFr0=vr0=vr0dc8meaabaqaciaacaGaaeqabaqabeGadaaakeaacqGG7bWEcuWGXbqCgaqeamaaBaaaleaacqaIXaqmaeqaaOGaeiilaWIaeiOla4IaeiOla4IaeiOla4IaeiilaWIafmyCaeNbaebadaWgaaWcbaGaem4AaS2aaSbaaWqaaiabdghaXbqabaaaleqaaOGaeiyFa0haaa@3B7C@. The *conditional entropy *[24] of *P *given *Q *is defined as follows.

H(P|Q)=∑j=1kqPr⁡(q¯j)H(P|q¯j)=−∑j=1kqPr⁡(q¯j)∑i=1kpPr⁡(p¯i|q¯j)log⁡Pr⁡(p¯i|q¯j)=−∑j=1kq∑i=1kpPr⁡(p¯i,q¯j)log⁡Pr⁡(p¯i|q¯j).
 MathType@MTEF@5@5@+=feaafiart1ev1aaatCvAUfKttLearuWrP9MDH5MBPbIqV92AaeXatLxBI9gBaebbnrfifHhDYfgasaacH8akY=wiFfYdH8Gipec8Eeeu0xXdbba9frFj0=OqFfea0dXdd9vqai=hGuQ8kuc9pgc9s8qqaq=dirpe0xb9q8qiLsFr0=vr0=vr0dc8meaabaqaciaacaGaaeqabaqabeGadaaakeaafaqaaeWadaaabaGaemisaGKaeiikaGIaemiuaaLaeiiFaWNaemyuaeLaeiykaKcabaGaeyypa0dabaWaaabCaeaacyGGqbaucqGGYbGCcqGGOaakcuWGXbqCgaqeamaaBaaaleaacqWGQbGAaeqaaOGaeiykaKIaemisaGKaeiikaGIaemiuaaLaeiiFaWNafmyCaeNbaebadaWgaaWcbaGaemOAaOgabeaakiabcMcaPaWcbaGaemOAaOMaeyypa0JaeGymaedabaGaem4AaS2aaSbaaWqaaiabdghaXbqabaaaniabggHiLdaakeaaaeaacqGH9aqpaeaacqGHsisldaaeWbqaaiGbccfaqjabckhaYjabcIcaOiqbdghaXzaaraWaaSbaaSqaaiabdQgaQbqabaGccqGGPaqkaSqaaiabdQgaQjabg2da9iabigdaXaqaaiabdUgaRnaaBaaameaacqWGXbqCaeqaaaqdcqGHris5aOWaaabCaeaacyGGqbaucqGGYbGCcqGGOaakcuWGWbaCgaqeamaaBaaaleaacqWGPbqAaeqaaOGaeiiFaWNafmyCaeNbaebadaWgaaWcbaGaemOAaOgabeaakiabcMcaPiGbcYgaSjabc+gaVjabcEgaNjGbccfaqjabckhaYjabcIcaOiqbdchaWzaaraWaaSbaaSqaaiabdMgaPbqabaGccqGG8baFcuWGXbqCgaqeamaaBaaaleaacqWGQbGAaeqaaOGaeiykaKcaleaacqWGPbqAcqGH9aqpcqaIXaqmaeaacqWGRbWAdaWgaaadbaGaemiCaahabeaaa0GaeyyeIuoaaOqaaaqaaiabg2da9aqaaiabgkHiTmaaqahabaWaaabCaeaacyGGqbaucqGGYbGCcqGGOaakcuWGWbaCgaqeamaaBaaaleaacqWGPbqAaeqaaOGaeiilaWIafmyCaeNbaebadaWgaaWcbaGaemOAaOgabeaakiabcMcaPiGbcYgaSjabc+gaVjabcEgaNjGbccfaqjabckhaYjabcIcaOiqbdchaWzaaraWaaSbaaSqaaiabdMgaPbqabaGccqGG8baFcuWGXbqCgaqeamaaBaaaleaacqWGQbGAaeqaaOGaeiykaKcaleaacqWGPbqAcqGH9aqpcqaIXaqmaeaacqWGRbWAdaWgaaadbaGaemiCaahabeaaa0GaeyyeIuoaaSqaaiabdQgaQjabg2da9iabigdaXaqaaiabdUgaRnaaBaaameaacqWGXbqCaeqaaaqdcqGHris5aOGaeiOla4caaaaa@B2AF@

That is, the conditional entropy of segmentation *P *given segmentation *Q *is the expected amount of information we need to identify the segment of *P *a point belongs to, given that we know the segment of this point in *Q*.

The following lemma gives an efficient algorithm for computing the conditional entropies between two segmentations. The algorithm runs in time *O *(*k*_*p *_+ *k*_*q*_).

**Lemma 1**. *Let P and Q be two segmentations. Denote by U their union, i.e., the segmentation defined by the segment boundaries that appear in P or in Q. The conditional entropy of P given Q, H (P|Q), can be computed using the following closed formula*

*H *(*P*|*Q*) = *H *(*U*) - *H *(*Q*).

*Proof*. Assume that segmentation *P *has *k*_*p *_segments {p¯1,...,p¯kp}
 MathType@MTEF@5@5@+=feaafiart1ev1aaatCvAUfKttLearuWrP9MDH5MBPbIqV92AaeXatLxBI9gBaebbnrfifHhDYfgasaacH8akY=wiFfYdH8Gipec8Eeeu0xXdbba9frFj0=OqFfea0dXdd9vqai=hGuQ8kuc9pgc9s8qqaq=dirpe0xb9q8qiLsFr0=vr0=vr0dc8meaabaqaciaacaGaaeqabaqabeGadaaakeaacqGG7bWEcuWGWbaCgaqeamaaBaaaleaacqaIXaqmaeqaaOGaeiilaWIaeiOla4IaeiOla4IaeiOla4IaeiilaWIafmiCaaNbaebadaWgaaWcbaGaem4AaS2aaSbaaWqaaiabdchaWbqabaaaleqaaOGaeiyFa0haaa@3B76@ and segmentation *Q *has *k*_*q *_segments {q¯1,...,q¯kq}
 MathType@MTEF@5@5@+=feaafiart1ev1aaatCvAUfKttLearuWrP9MDH5MBPbIqV92AaeXatLxBI9gBaebbnrfifHhDYfgasaacH8akY=wiFfYdH8Gipec8Eeeu0xXdbba9frFj0=OqFfea0dXdd9vqai=hGuQ8kuc9pgc9s8qqaq=dirpe0xb9q8qiLsFr0=vr0=vr0dc8meaabaqaciaacaGaaeqabaqabeGadaaakeaacqGG7bWEcuWGXbqCgaqeamaaBaaaleaacqaIXaqmaeqaaOGaeiilaWIaeiOla4IaeiOla4IaeiOla4IaeiilaWIafmyCaeNbaebadaWgaaWcbaGaem4AaS2aaSbaaWqaaiabdghaXbqabaaaleqaaOGaeiyFa0haaa@3B7C@. Using Equation (1) we can obtain the desired result. That is,

H(P|Q)=−∑i=1kp∑j=1kqPr⁡(p¯i,q¯j)log⁡(Pr⁡(p¯i|q¯j))=−∑i=1kp∑j=1kqPr⁡(p¯i,q¯j)log⁡(Pr⁡(p¯i,q¯j))+∑i=1kp∑j=1kqPr⁡(p¯i,q¯j)log⁡(Pr⁡(q¯j))=H(U)+∑j=1kqlog⁡(Pr⁡(q¯j))∑i=1kpPr⁡(p¯i,q¯j)=H(U)+∑j=1kqlog⁡(Pr⁡(q¯j))Pr⁡(q¯j)=H(U)−H(Q).
 MathType@MTEF@5@5@+=feaafiart1ev1aaatCvAUfKttLearuWrP9MDH5MBPbIqV92AaeXatLxBI9gBaebbnrfifHhDYfgasaacH8akY=wiFfYdH8Gipec8Eeeu0xXdbba9frFj0=OqFfea0dXdd9vqai=hGuQ8kuc9pgc9s8qqaq=dirpe0xb9q8qiLsFr0=vr0=vr0dc8meaabaqaciaacaGaaeqabaqabeGadaaakeaafaqaaeGbdaaaaeaacqWGibascqGGOaakcqWGqbaucqGG8baFcqWGrbqucqGGPaqkaeaacqGH9aqpaeaacqGHsisldaaeWbqaamaaqahabaGagiiuaaLaeiOCaiNaeiikaGIafmiCaaNbaebadaWgaaWcbaGaemyAaKgabeaakiabcYcaSiqbdghaXzaaraWaaSbaaSqaaiabdQgaQbqabaGccqGGPaqkcyGGSbaBcqGGVbWBcqGGNbWzcqGGOaakcyGGqbaucqGGYbGCcqGGOaakcuWGWbaCgaqeamaaBaaaleaacqWGPbqAaeqaaOGaeiiFaWNafmyCaeNbaebadaWgaaWcbaGaemOAaOgabeaakiabcMcaPiabcMcaPaWcbaGaemOAaOMaeyypa0JaeGymaedabaGaem4AaS2aaSbaaWqaaiabdghaXbqabaaaniabggHiLdaaleaacqWGPbqAcqGH9aqpcqaIXaqmaeaacqWGRbWAdaWgaaadbaGaemiCaahabeaaa0GaeyyeIuoaaOqaaaqaaiabg2da9aqaaiabgkHiTmaaqahabaWaaabCaeaacyGGqbaucqGGYbGCcqGGOaakcuWGWbaCgaqeamaaBaaaleaacqWGPbqAaeqaaOGaeiilaWIafmyCaeNbaebadaWgaaWcbaGaemOAaOgabeaakiabcMcaPiGbcYgaSjabc+gaVjabcEgaNjabcIcaOiGbccfaqjabckhaYjabcIcaOiqbdchaWzaaraWaaSbaaSqaaiabdMgaPbqabaGccqGGSaalcuWGXbqCgaqeamaaBaaaleaacqWGQbGAaeqaaOGaeiykaKIaeiykaKcaleaacqWGQbGAcqGH9aqpcqaIXaqmaeaacqWGRbWAdaWgaaadbaGaemyCaehabeaaa0GaeyyeIuoaaSqaaiabdMgaPjabg2da9iabigdaXaqaaiabdUgaRnaaBaaameaacqWGWbaCaeqaaaqdcqGHris5aaGcbaaabaaabaGaey4kaSYaaabCaeaadaaeWbqaaiGbccfaqjabckhaYjabcIcaOiqbdchaWzaaraWaaSbaaSqaaiabdMgaPbqabaGccqGGSaalcuWGXbqCgaqeamaaBaaaleaacqWGQbGAaeqaaOGaeiykaKIagiiBaWMaei4Ba8Maei4zaCMaeiikaGIagiiuaaLaeiOCaiNaeiikaGIafmyCaeNbaebadaWgaaWcbaGaemOAaOgabeaakiabcMcaPiabcMcaPaWcbaGaemOAaOMaeyypa0JaeGymaedabaGaem4AaS2aaSbaaWqaaiabdghaXbqabaaaniabggHiLdaaleaacqWGPbqAcqGH9aqpcqaIXaqmaeaacqWGRbWAdaWgaaadbaGaemiCaahabeaaa0GaeyyeIuoaaOqaaaqaaiabg2da9aqaaiabdIeaijabcIcaOiabdwfavjabcMcaPiabgUcaRmaaqahabaGagiiBaWMaei4Ba8Maei4zaCMaeiikaGIagiiuaaLaeiOCaiNaeiikaGIafmyCaeNbaebadaWgaaWcbaGaemOAaOgabeaakiabcMcaPiabcMcaPaWcbaGaemOAaOMaeyypa0JaeGymaedabaGaem4AaS2aaSbaaWqaaiabdghaXbqabaaaniabggHiLdGcdaaeWbqaaiGbccfaqjabckhaYjabcIcaOiqbdchaWzaaraWaaSbaaSqaaiabdMgaPbqabaGccqGGSaalcuWGXbqCgaqeamaaBaaaleaacqWGQbGAaeqaaOGaeiykaKcaleaacqWGPbqAcqGH9aqpcqaIXaqmaeaacqWGRbWAdaWgaaadbaGaemiCaahabeaaa0GaeyyeIuoaaOqaaaqaaiabg2da9aqaaiabdIeaijabcIcaOiabdwfavjabcMcaPiabgUcaRmaaqahabaGagiiBaWMaei4Ba8Maei4zaCMaeiikaGIagiiuaaLaeiOCaiNaeiikaGIafmyCaeNbaebadaWgaaWcbaGaemOAaOgabeaakiabcMcaPiabcMcaPiGbccfaqjabckhaYjabcIcaOiqbdghaXzaaraWaaSbaaSqaaiabdQgaQbqabaGccqGGPaqkaSqaaiabdQgaQjabg2da9iabigdaXaqaaiabdUgaRnaaBaaameaacqWGXbqCaeqaaaqdcqGHris5aaGcbaaabaGaeyypa0dabaGaemisaGKaeiikaGIaemyvauLaeiykaKIaeyOeI0IaemisaGKaeiikaGIaemyuaeLaeiykaKIaeiOla4caaaaa@1AEC@

Intuitively, the entropy of *P *given *Q *tells us how much information we obtain about *P*, if we know that that we are in a specific segment of *Q*. The more information *Q *reveals about the structure *P*, the more *similar *segmentations *P *and *Q *are, and the smaller the value of *H *(*P*|*Q*).

The single value *H *(*P*|*Q*) does not give the whole picture of segmentation similarity, however. For example, consider the case where segmentation *Q *consists of a single segment. Then, using Lemma 1 we can verify our intuition that knowledge about *Q *gives us no information about *P*, i.e., *H *(*P*|*Q*) = *H *(*P*). However, notice that *H *(*Q*|*P*) = 0, for any *P*.

Consider also the case where *Q *consists of *N *segments where each segment has length 1. In this case, *H *(*P*|*Q*) = 0, that is, *Q *gives lots of information about *P*, irrespective of the structure of *P*. As before, observe that *H *(*Q*|*P*) = log *N *- *H *(*P*). Thus, if *P *has low entropy, the value of *H *(*Q*|*P*) is large.

The above examples show that the similarity of two segmentations *P *and *Q *cannot be judged just by the single value *H *(*P*|*Q*) or *H *(*Q*|*P*). Rather, we can conclude that segmentations *P *and *Q *are similar only if both *H *(*P*|*Q*) and *H *(*Q*|*P*) are small. Even when using the *entropy distance *between two segmentations (see [25]), defined as *D*_*H *_(*P*, *Q*) = *H *(*P*|*Q*) + *H *(*Q*|*P*), we can get small values to segmentations that are quite different. We show in the experimental section that considering the two conditional entropies separately gives more accurate results than using their sum.

### Randomization techniques

Consider a segmentation algorithm that given as input sequence *S *outputs a segmentation *P*. The plethora of segmentation algorithms and segmentation criteria naturally raises the question of how good and how informative segmentation *P *is. Assume that we a priori know a ground-truth segmentation *T *of *S*. Then, we can say that segmentation *P *is good if *P *is similar to *T*. Thus, using the definitions in the Methods section, *P *is a good segmentation if *H *(*P*|*T*) and *H *(*T*|*P*) are small. However, a natural question is how small is small enough? Or, is there a threshold in the values of the conditional entropies below which we can characterize segmentation *P *as being correct or interesting? Finally, can we set this threshold universally for all segmentations? In this section we describe a set of randomization techniques that we devise in order to provide an answer to these questions.

Our generic methodology is the following. Given a segmentation *P *and a ground-truth segmentation *T *of the same sequence, we first compute *H *(*P*|*T*) and *H *(*T*|*P*). We compare the values of these conditional entropies with the values of the conditional entropies *H *(*R*|*T*) and *H *(*T*|*R*) for a random segmentation *R*. We conclude that *P *is similar to *T*, and thus interesting, if the values of *H *(*P*|*T*) (and *H *(*T*|*P*)) are small compared to the values of *H *(*R*|*T*) (and *H *(*T*|*R*)) for a large majority of random segmentations *R*.

Consider a class CN
 MathType@MTEF@5@5@+=feaafiart1ev1aaatCvAUfKttLearuWrP9MDH5MBPbIqV92AaeXatLxBI9gBaebbnrfifHhDYfgasaacH8akY=wiFfYdH8Gipec8Eeeu0xXdbba9frFj0=OqFfea0dXdd9vqai=hGuQ8kuc9pgc9s8qqaq=dirpe0xb9q8qiLsFr0=vr0=vr0dc8meaabaqaciaacaGaaeqabaqabeGadaaakeaaruWrPXgBtfMBZbaceaGae83qam0aaSbaaSqaaGqaciab+5eaobqabaaaaa@324D@ of segmentations for sequences of length *N*. Then, the randomization test is conducted as follows. Pick random segmentations *R *∈ CN
 MathType@MTEF@5@5@+=feaafiart1ev1aaatCvAUfKttLearuWrP9MDH5MBPbIqV92AaeXatLxBI9gBaebbnrfifHhDYfgasaacH8akY=wiFfYdH8Gipec8Eeeu0xXdbba9frFj0=OqFfea0dXdd9vqai=hGuQ8kuc9pgc9s8qqaq=dirpe0xb9q8qiLsFr0=vr0=vr0dc8meaabaqaciaacaGaaeqabaqabeGadaaakeaaruWrPXgBtfMBZbaceaGae83qam0aaSbaaSqaaGqaciab+5eaobqabaaaaa@324D@. For each such *R *compute *H *(*T*|*R*), and compare *H *(*T*|*P*) against the distribution of the values *H *(*T*|*R*). Similarly, compute the values of *H *(*R*|*T*) for a large number of segmentations *R *∈ CN
 MathType@MTEF@5@5@+=feaafiart1ev1aaatCvAUfKttLearuWrP9MDH5MBPbIqV92AaeXatLxBI9gBaebbnrfifHhDYfgasaacH8akY=wiFfYdH8Gipec8Eeeu0xXdbba9frFj0=OqFfea0dXdd9vqai=hGuQ8kuc9pgc9s8qqaq=dirpe0xb9q8qiLsFr0=vr0=vr0dc8meaabaqaciaacaGaaeqabaqabeGadaaakeaaruWrPXgBtfMBZbaceaGae83qam0aaSbaaSqaaGqaciab+5eaobqabaaaaa@324D@ and compare these values with the value of *H *(*P*|*T*). In general, if segmentations *T *and *P *have a very different number of of segments, one of *H *(*T*|*R*) and *H*(*R*|*T*) will be large for any *R *from CN
 MathType@MTEF@5@5@+=feaafiart1ev1aaatCvAUfKttLearuWrP9MDH5MBPbIqV92AaeXatLxBI9gBaebbnrfifHhDYfgasaacH8akY=wiFfYdH8Gipec8Eeeu0xXdbba9frFj0=OqFfea0dXdd9vqai=hGuQ8kuc9pgc9s8qqaq=dirpe0xb9q8qiLsFr0=vr0=vr0dc8meaabaqaciaacaGaaeqabaqabeGadaaakeaaruWrPXgBtfMBZbaceaGae83qam0aaSbaaSqaaGqaciab+5eaobqabaaaaa@324D@. The randomization method we describe is best suited for the case when *T *and *P *have about the same number of segments.

We still need to specify the class CN
 MathType@MTEF@5@5@+=feaafiart1ev1aaatCvAUfKttLearuWrP9MDH5MBPbIqV92AaeXatLxBI9gBaebbnrfifHhDYfgasaacH8akY=wiFfYdH8Gipec8Eeeu0xXdbba9frFj0=OqFfea0dXdd9vqai=hGuQ8kuc9pgc9s8qqaq=dirpe0xb9q8qiLsFr0=vr0=vr0dc8meaabaqaciaacaGaaeqabaqabeGadaaakeaaruWrPXgBtfMBZbaceaGae83qam0aaSbaaSqaaGqaciab+5eaobqabaaaaa@324D@ of segmentations from which the random segmentations are picked. We define two classes of segmentations. Intuitively, the first class is used for checking if the candidate segmentation *P *is significantly closer to *T *than random segmentations *R *with the same number of segments as in *T*. Imagine that the segmentation procedure that generated *P *has knowledge of the segment number in *T*. By using this class we find out if it is enough to guess a segmentation as close to *T *as *P *is, by just randomly assigning a correct number of segment boundaries. The other class is used similarly, for checking if the knowledge of *T*'s segment length distribution is enough to generate segmentations as close to *T *as *P*. An analog is found in classification problems, where the true class labels in *T *are permuted to check if the candidate classification *P *offers more insight into *T *than we would expect from guessing a random classification *R*.

In the first case, if *T *has *k *segments, then we restrict the random segmentations to those that have *k *segments as well. We denote by CN,k
 MathType@MTEF@5@5@+=feaafiart1ev1aaatCvAUfKttLearuWrP9MDH5MBPbIqV92AaeXatLxBI9gBaebbnrfifHhDYfgasaacH8akY=wiFfYdH8Gipec8Eeeu0xXdbba9frFj0=OqFfea0dXdd9vqai=hGuQ8kuc9pgc9s8qqaq=dirpe0xb9q8qiLsFr0=vr0=vr0dc8meaabaqaciaacaGaaeqabaqabeGadaaakeaaruWrPXgBtfMBZbaceaGae83qam0aaSbaaSqaaGqaciab+5eaoHqaaiab9XcaSiab+TgaRbqabaaaaa@348B@ the class of all segmentations with *k *segments that partition sequences of length *N*. We have |CN,k|=(N(k−1))
 MathType@MTEF@5@5@+=feaafiart1ev1aaatCvAUfKttLearuWrP9MDH5MBPbIqV92AaeXatLxBI9gBaebbnrfifHhDYfgasaacH8akY=wiFfYdH8Gipec8Eeeu0xXdbba9frFj0=OqFfea0dXdd9vqai=hGuQ8kuc9pgc9s8qqaq=dirpe0xb9q8qiLsFr0=vr0=vr0dc8meaabaqaciaacaGaaeqabaqabeGadaaakeaaiiaacqWF8baFruWrPXgBtfMBZbaceaGae43qam0aaSbaaSqaaGqaciab95eaojab9XcaSiab9TgaRbqabaGccqWF8baFcqGH9aqpdaqadaqaauaabeqaceaaaeaacqWGobGtaeaacqGGOaakcqWGRbWAcqGHsislcqaIXaqmcqGGPaqkaaaacaGLOaGaayzkaaaaaa@403C@, since there are (n(k−1))
 MathType@MTEF@5@5@+=feaafiart1ev1aaatCvAUfKttLearuWrP9MDH5MBPbIqV92AaeXatLxBI9gBaebbnrfifHhDYfgasaacH8akY=wiFfYdH8Gipec8Eeeu0xXdbba9frFj0=OqFfea0dXdd9vqai=hGuQ8kuc9pgc9s8qqaq=dirpe0xb9q8qiLsFr0=vr0=vr0dc8meaabaqaciaacaGaaeqabaqabeGadaaakeaadaqadaqaauaabeqaceaaaeaacqWGUbGBaeaacqGGOaakcqWGRbWAcqGHsislcqaIXaqmcqGGPaqkaaaacaGLOaGaayzkaaaaaa@3495@ ways to choose *k *- 1 segment boundaries from the *N *points of the sequence (the first and the last boundary are always fixed). We call the randomization test in which the random segmentations *R *∈ CN,k
 MathType@MTEF@5@5@+=feaafiart1ev1aaatCvAUfKttLearuWrP9MDH5MBPbIqV92AaeXatLxBI9gBaebbnrfifHhDYfgasaacH8akY=wiFfYdH8Gipec8Eeeu0xXdbba9frFj0=OqFfea0dXdd9vqai=hGuQ8kuc9pgc9s8qqaq=dirpe0xb9q8qiLsFr0=vr0=vr0dc8meaabaqaciaacaGaaeqabaqabeGadaaakeaaruWrPXgBtfMBZbaceaGae83qam0aaSbaaSqaaGqaciab+5eaoHqaaiab9XcaSiab+TgaRbqabaaaaa@348B@ a *k-randomization test*.

We also introduce the *ℓ-randomization test*, specified as follows. Consider a segmentation *T *with segments {t¯1,...,t¯k}
 MathType@MTEF@5@5@+=feaafiart1ev1aaatCvAUfKttLearuWrP9MDH5MBPbIqV92AaeXatLxBI9gBaebbnrfifHhDYfgasaacH8akY=wiFfYdH8Gipec8Eeeu0xXdbba9frFj0=OqFfea0dXdd9vqai=hGuQ8kuc9pgc9s8qqaq=dirpe0xb9q8qiLsFr0=vr0=vr0dc8meaabaqaciaacaGaaeqabaqabeGadaaakeaacqGG7bWEcuWG0baDgaqeamaaBaaaleaacqaIXaqmaeqaaOGaeiilaWIaeiOla4IaeiOla4IaeiOla4IaeiilaWIafmiDaqNbaebadaWgaaWcbaGaem4AaSgabeaakiabc2ha9baa@39E5@. Each segment t¯i
 MathType@MTEF@5@5@+=feaafiart1ev1aaatCvAUfKttLearuWrP9MDH5MBPbIqV92AaeXatLxBI9gBaebbnrfifHhDYfgasaacH8akY=wiFfYdH8Gipec8Eeeu0xXdbba9frFj0=OqFfea0dXdd9vqai=hGuQ8kuc9pgc9s8qqaq=dirpe0xb9q8qiLsFr0=vr0=vr0dc8meaabaqaciaacaGaaeqabaqabeGadaaakeaacuWG0baDgaqeamaaBaaaleaacqWGPbqAaeqaaaaa@2FBC@ has length |t¯i
 MathType@MTEF@5@5@+=feaafiart1ev1aaatCvAUfKttLearuWrP9MDH5MBPbIqV92AaeXatLxBI9gBaebbnrfifHhDYfgasaacH8akY=wiFfYdH8Gipec8Eeeu0xXdbba9frFj0=OqFfea0dXdd9vqai=hGuQ8kuc9pgc9s8qqaq=dirpe0xb9q8qiLsFr0=vr0=vr0dc8meaabaqaciaacaGaaeqabaqabeGadaaakeaacuWG0baDgaqeamaaBaaaleaacqWGPbqAaeqaaaaa@2FBC@| and these lengths define a distribution of the segment lengths of the segmentation. There are a total of at most *k*! segmentations that have the same segment lengths as *T *does (maximized when all segments in *T *have different lengths). For a given distribution of segment lengths *ℓ *we denote by CN,k,ℓ
 MathType@MTEF@5@5@+=feaafiart1ev1aaatCvAUfKttLearuWrP9MDH5MBPbIqV92AaeXatLxBI9gBaebbnrfifHhDYfgasaacH8akY=wiFfYdH8Gipec8Eeeu0xXdbba9frFj0=OqFfea0dXdd9vqai=hGuQ8kuc9pgc9s8qqaq=dirpe0xb9q8qiLsFr0=vr0=vr0dc8meaabaqaciaacaGaaeqabaqabeGadaaakeaaruWrPXgBtfMBZbaceaGae83qam0aaSbaaSqaaGqaciab+5eaoHqaaiab9XcaSiab+TgaRjab+XcaSiabloriSbqabaaaaa@3698@ the class of segmentations with *k *segments and lengths *ℓ*. Obviously, CN,k,ℓ
 MathType@MTEF@5@5@+=feaafiart1ev1aaatCvAUfKttLearuWrP9MDH5MBPbIqV92AaeXatLxBI9gBaebbnrfifHhDYfgasaacH8akY=wiFfYdH8Gipec8Eeeu0xXdbba9frFj0=OqFfea0dXdd9vqai=hGuQ8kuc9pgc9s8qqaq=dirpe0xb9q8qiLsFr0=vr0=vr0dc8meaabaqaciaacaGaaeqabaqabeGadaaakeaaruWrPXgBtfMBZbaceaGae83qam0aaSbaaSqaaGqaciab+5eaoHqaaiab9XcaSiab+TgaRjab+XcaSiabloriSbqabaaaaa@3698@ ⊂ CN,k
 MathType@MTEF@5@5@+=feaafiart1ev1aaatCvAUfKttLearuWrP9MDH5MBPbIqV92AaeXatLxBI9gBaebbnrfifHhDYfgasaacH8akY=wiFfYdH8Gipec8Eeeu0xXdbba9frFj0=OqFfea0dXdd9vqai=hGuQ8kuc9pgc9s8qqaq=dirpe0xb9q8qiLsFr0=vr0=vr0dc8meaabaqaciaacaGaaeqabaqabeGadaaakeaaruWrPXgBtfMBZbaceaGae83qam0aaSbaaSqaaGqaciab+5eaoHqaaiab9XcaSiab+TgaRbqabaaaaa@348B@ and CN,k,ℓ
 MathType@MTEF@5@5@+=feaafiart1ev1aaatCvAUfKttLearuWrP9MDH5MBPbIqV92AaeXatLxBI9gBaebbnrfifHhDYfgasaacH8akY=wiFfYdH8Gipec8Eeeu0xXdbba9frFj0=OqFfea0dXdd9vqai=hGuQ8kuc9pgc9s8qqaq=dirpe0xb9q8qiLsFr0=vr0=vr0dc8meaabaqaciaacaGaaeqabaqabeGadaaakeaaruWrPXgBtfMBZbaceaGae83qam0aaSbaaSqaaGqaciab+5eaoHqaaiab9XcaSiab+TgaRjab+XcaSiabloriSbqabaaaaa@3698@ ≤ *k*!, since the segmentations in CN,k,ℓ
 MathType@MTEF@5@5@+=feaafiart1ev1aaatCvAUfKttLearuWrP9MDH5MBPbIqV92AaeXatLxBI9gBaebbnrfifHhDYfgasaacH8akY=wiFfYdH8Gipec8Eeeu0xXdbba9frFj0=OqFfea0dXdd9vqai=hGuQ8kuc9pgc9s8qqaq=dirpe0xb9q8qiLsFr0=vr0=vr0dc8meaabaqaciaacaGaaeqabaqabeGadaaakeaaruWrPXgBtfMBZbaceaGae83qam0aaSbaaSqaaGqaciab+5eaoHqaaiab9XcaSiab+TgaRjab+XcaSiabloriSbqabaaaaa@3698@. differ only in the order in which the segments with different lengths appear. Note that for a random segmentation *R *∈ CN,k,ℓ
 MathType@MTEF@5@5@+=feaafiart1ev1aaatCvAUfKttLearuWrP9MDH5MBPbIqV92AaeXatLxBI9gBaebbnrfifHhDYfgasaacH8akY=wiFfYdH8Gipec8Eeeu0xXdbba9frFj0=OqFfea0dXdd9vqai=hGuQ8kuc9pgc9s8qqaq=dirpe0xb9q8qiLsFr0=vr0=vr0dc8meaabaqaciaacaGaaeqabaqabeGadaaakeaaruWrPXgBtfMBZbaceaGae83qam0aaSbaaSqaaGqaciab+5eaoHqaaiab9XcaSiab+TgaRjab+XcaSiabloriSbqabaaaaa@3698@, the conditional entropies w.r.t. segmentation *T *are equal, i.e., *H *(*T*|*R*) = *H *(*R*|*T*). This follows from the fact that *H *(*T*) = *H *(*R*), since both segmentations contain exactly the same segments. The *ℓ*-randomization restricts our attention to random segmentations with segment length distribution being the same as the segment-length distribution of the ground-truth segmentation *T*. That is, the significance of *H *(*T*|*P*) and *H *(*P*|*T*) for a candidate segmentation *P *are evaluated under the assumption that the segment-length distribution is known. In the special case where all segments in *T *have the same length, the segment length distribution uniquely characterizes the segmentations *R *∈ CN,k,ℓ
 MathType@MTEF@5@5@+=feaafiart1ev1aaatCvAUfKttLearuWrP9MDH5MBPbIqV92AaeXatLxBI9gBaebbnrfifHhDYfgasaacH8akY=wiFfYdH8Gipec8Eeeu0xXdbba9frFj0=OqFfea0dXdd9vqai=hGuQ8kuc9pgc9s8qqaq=dirpe0xb9q8qiLsFr0=vr0=vr0dc8meaabaqaciaacaGaaeqabaqabeGadaaakeaaruWrPXgBtfMBZbaceaGae83qam0aaSbaaSqaaGqaciab+5eaoHqaaiab9XcaSiab+TgaRjab+XcaSiabloriSbqabaaaaa@3698@. In this case *H *(*T*|*R*) = *H* (*R*|*T*) = 0. Moreover, any segmentation *P *that does not have equal-length segments has *H *(*P*|*T*) > 0 and *H *(*T*|*P*) > 0 and thus is considered far from the ground truth w.r.t. the *ℓ*-randomization test.

## Authors' contributions

All authors participated in the design of the study and provided significant contributions to the content.

All authors were involved in writing and revising the manuscript.

## Supplementary Material

Additional file 1 **Randomization results for chromosome 1 isochore segmentations**. Randomization results for the isochore structure from [7] for a 100 Mb region of chromosome 1 with 1305 segments. Click here for file

Additional file 2 **Randomization results for MHC isochore segmentations**. Randomization results for the MHC region in chromosome 6 are shown w.r.t. three alternative ground truth segmentations *T *from [14,20,23]. Click here for file
