# Supplementary material for: Comparing segmentations by applying randomization techniques
Source: BMC Bioinformatics. 2007 May 23;8:171. doi: 10.1186/1471-2105-8-171 (PMC1904250; doi:10.1186/1471-2105-8-171)
Supplement: Additional file 1 — Randomization results for chromosome 1 isochore segmentations. Randomization results for the isochore structure from [7] for a 100 Mb region of chromosome 1 with 1305 segments. [file 1471-2105-8-171-S1.pdf]

Additional file 1 — Randomization results for chromosome 1 isochore segmentations

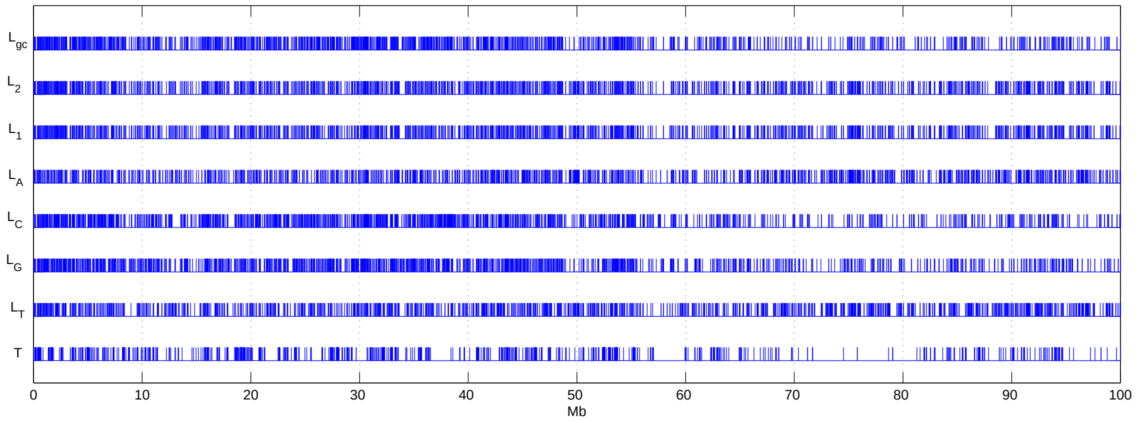

Figure 1: Isochore segmentations of Chromosome 1 100 Mb region with 1305 segments and reference segmentation T from [7]. L_f: least-squares segmentation with features f; $f \in \{gc, 2, 1, A, C, G, T\}$ indicate frequencies of G+C, 2-letter words, 1-letter words, and frequency of A, C, G, T, or G+C, respectively.

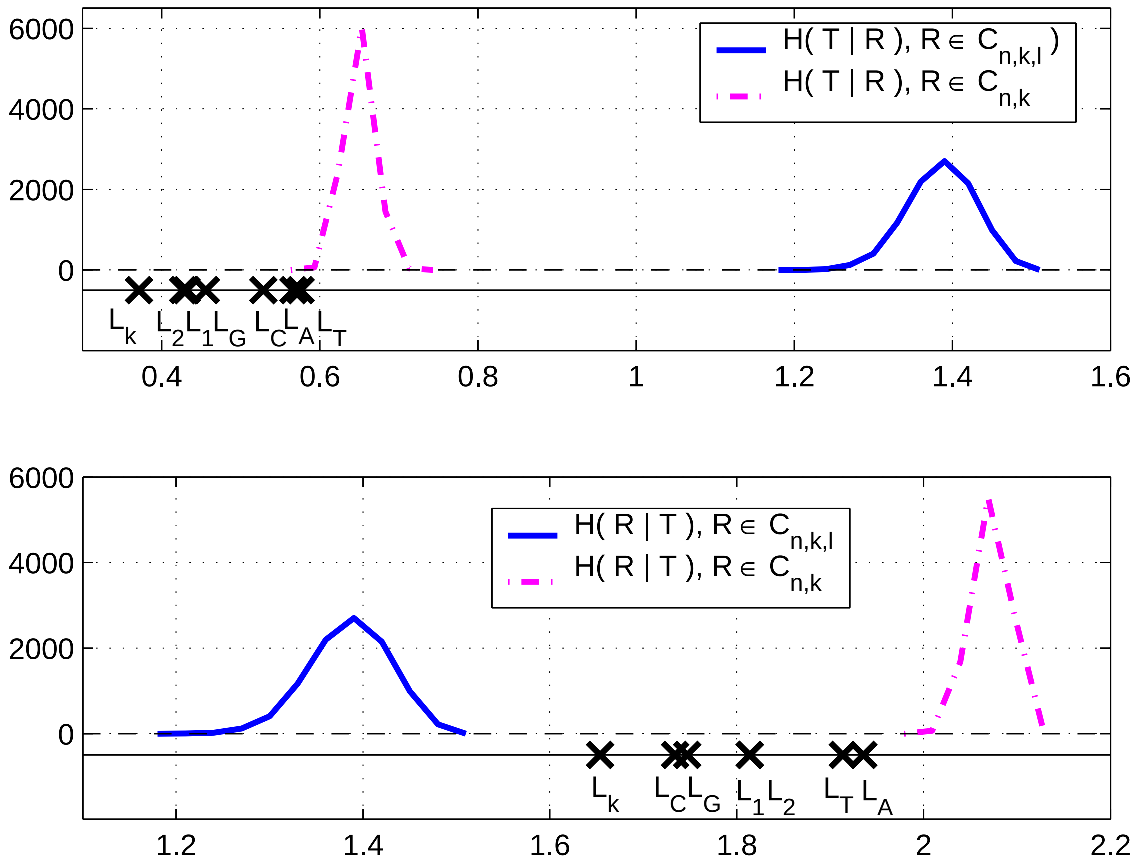

Figure 2: Randomization of Chromosome 1 isochore segmentations with 1305 segments: conditional entropies. L_f: least-squares segmentation with features f; $f \in \{gc, 2, 1, A, C, G, T\}$ indicate frequencies of G+C, 2-letter words, 1-letter words, and frequency of A, C, G, or T respectively.
