# Supplementary material for: Comparing segmentations by applying randomization techniques
Source: BMC Bioinformatics. 2007 May 23;8:171. doi: 10.1186/1471-2105-8-171 (PMC1904250; doi:10.1186/1471-2105-8-171)
Supplement: Additional file 2 — Randomization results for MHC isochore segmentations. Randomization results for the MHC region in chromosome 6 are shown w.r.t. three alternative ground truth segmentations T from [14,20,23]. [file 1471-2105-8-171-S2.pdf]

Additional file 2 — Randomization results for MHC isochore segmentations

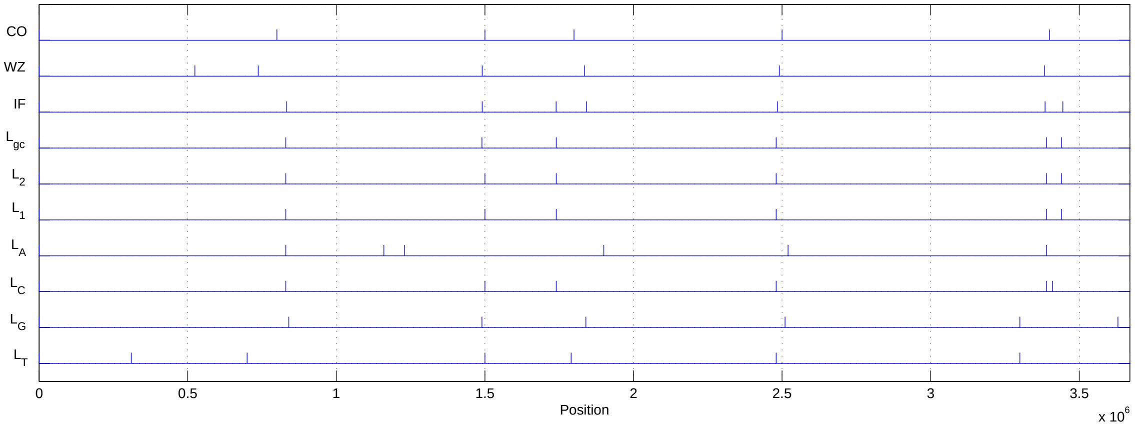

Figure 1: Segmentations of MHC region, segmentations WZ from [15], CO from [21], and IF from [24]. L_f: least-squares segmentation with features f; $f \in \{gc, 2, 1, A, C, G, T\}$ indicate frequencies of G+C, 2-letter words, 1-letter words, and frequency of A, C, G, T, or G+C, respectively.

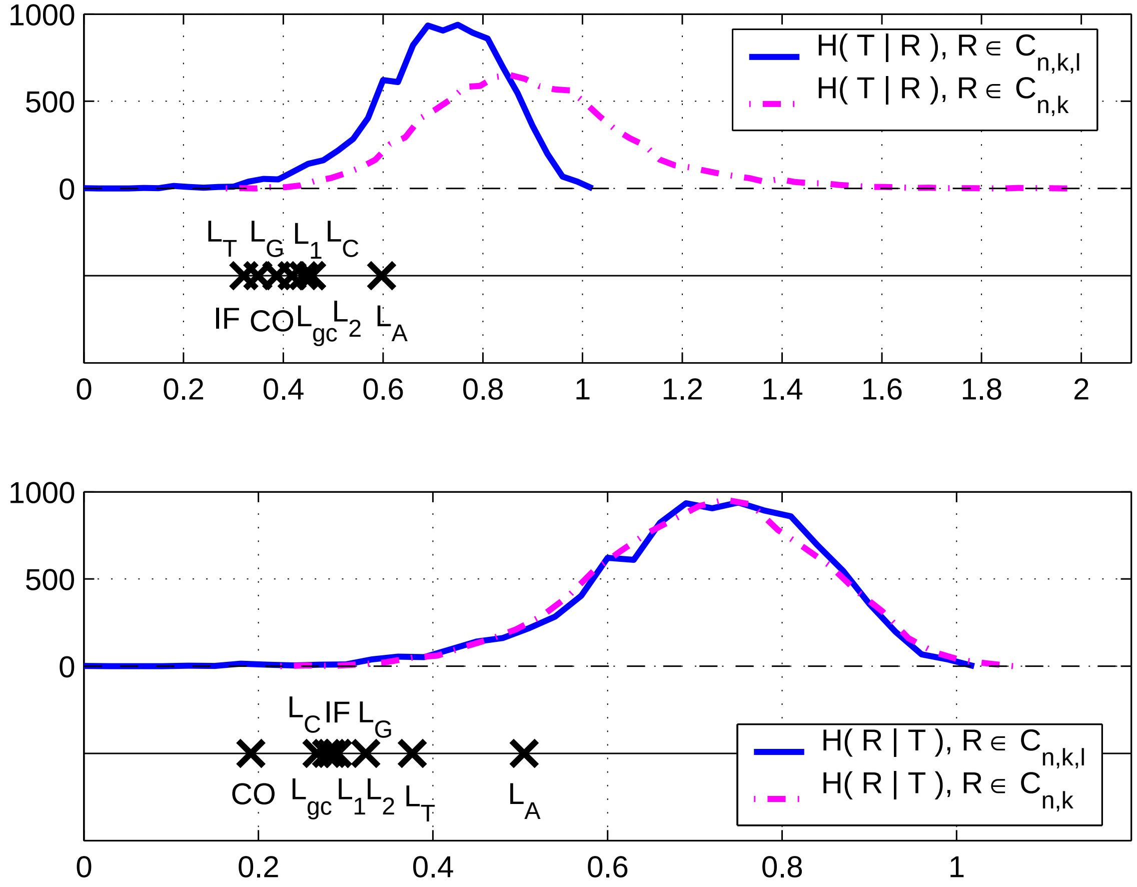

Figure 2: Randomization of MHC segmentations w.r.t. the ground truth from [15]: conditional entropies. Segmentations CO from [21], and IF from [24]. L_f: least-squares segmentation with features f; $f \in \{gc, 2, 1, A, C, G, T\}$ indicate frequencies of G+C, 2-letter words, 1-letter words, and frequency of A, C, G, or T respectively.

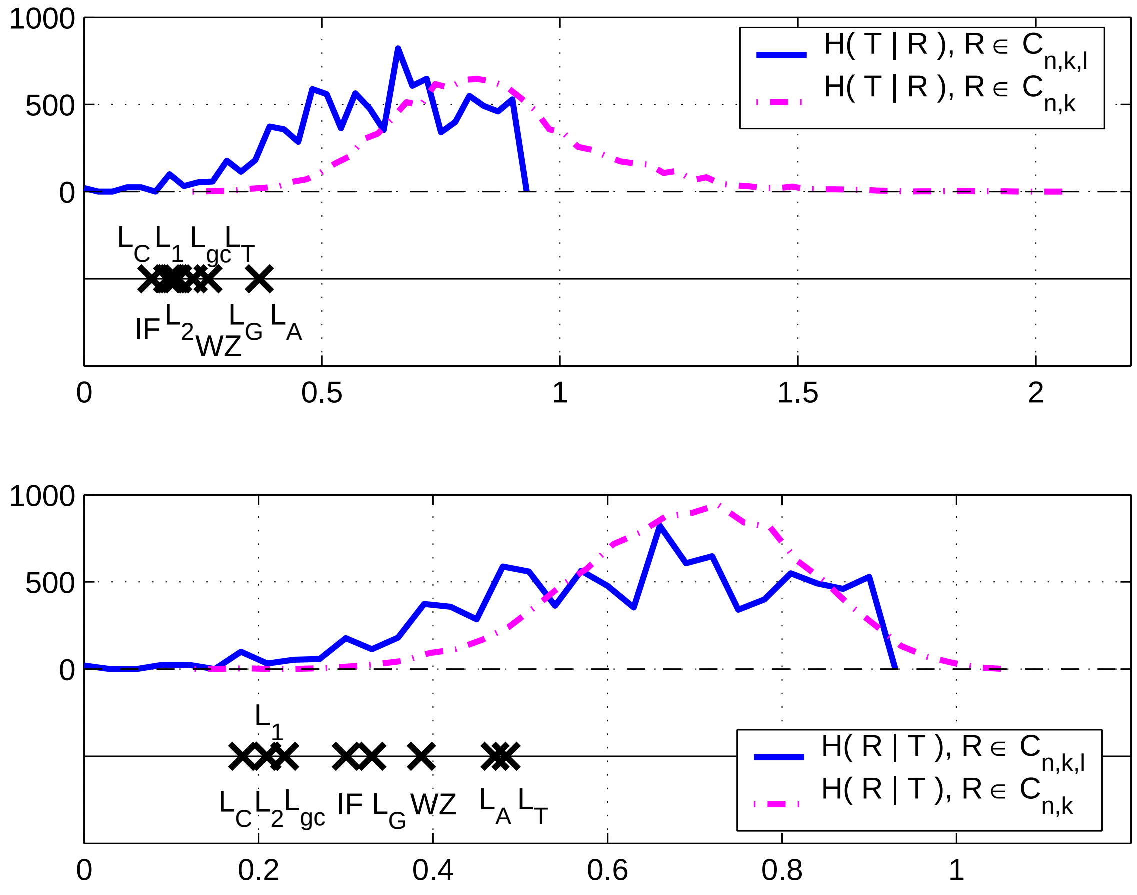

Figure 3: Randomization of MHC segmentations w.r.t. the ground truth from [21]: conditional entropies. Segmentations WZ from [15], and IF from [24]. L_f: least-squares segmentation with features f; $f \in \{gc, 2, 1, A, C, G, T\}$ indicate frequencies of G+C, 2-letter words, 1-letter words, and frequency of A, C, G, or T respectively.

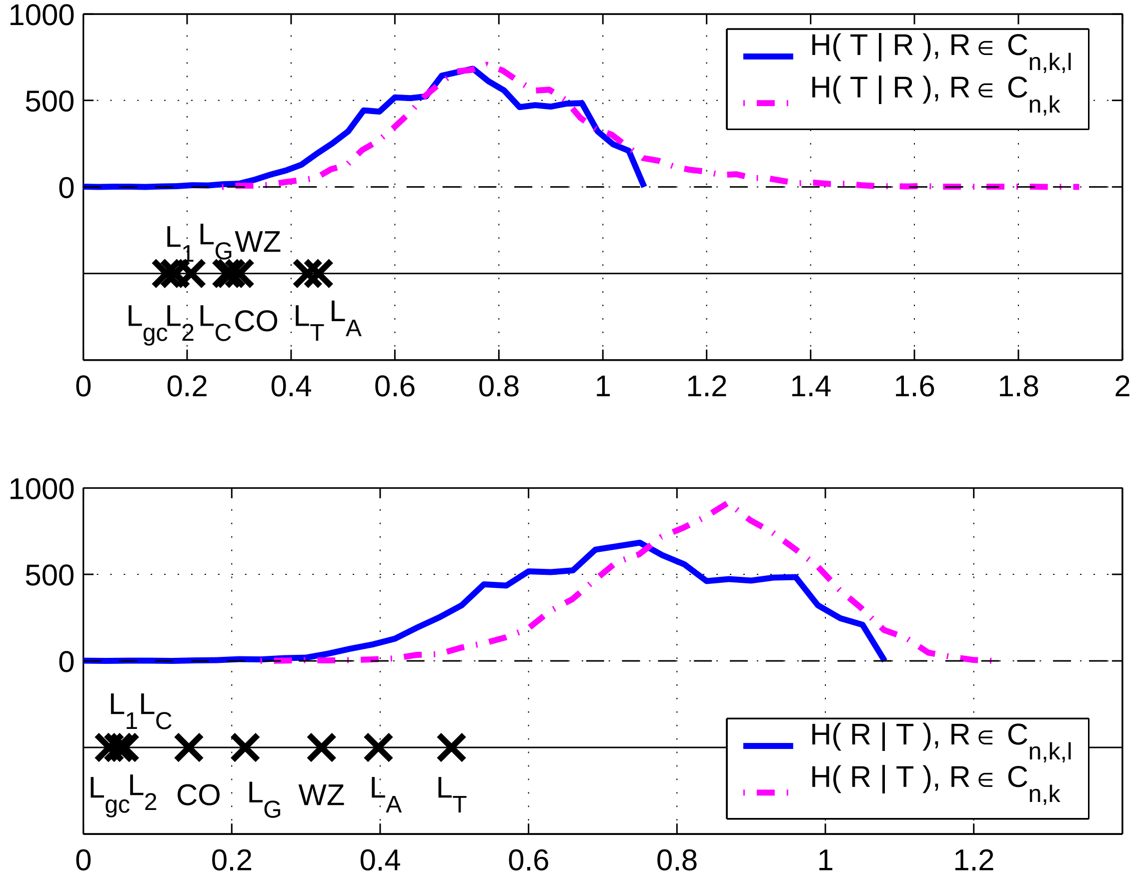

Figure 4: Randomization of MHC segmentations w.r.t. the ground truth from [24]:conditional entropies. Segmentations WZ from [15], and CO from [21]. L_f: least-squares segmentation with features f; $f \in \{gc, 2, 1, A, C, G, T\}$ indicate frequencies of G+C, 2-letter words, 1-letter words, and frequency of A, C, G, or T respectively.
